# Factors associated with gilt retention in breeding herds under one production system in the Midwestern United States

**DOI:** 10.1093/tas/txag168

**Published:** 2026-10-22

**Authors:** Elly Kirwa, Ben Haberl, Swaminathan Jayaraman, Rafael da Rosa Ulguim, Gustavo S Silva

**Affiliations:** Veterinary Diagnostic and Production Animal Medicine Department, Iowa State University, Ames, IA, United States; Iowa Select Farms, Iowa Falls, IA, United States; Veterinary Diagnostic and Production Animal Medicine Department, Iowa State University, Ames, IA, United States; Animal Medicine Department, Federal University of Rio Grande do Sul, Porto Alegre, Brazil; Veterinary Diagnostic and Production Animal Medicine Department, Iowa State University, Ames, IA, United States

**Keywords:** gilt retention, sow removal, age at first service, PRRSV lifetime status

## Abstract

Sow longevity and lifetime productivity are critical determinants of breeding herd profitability and welfare. However, many gilts are removed before parity 3, with 15% to 20% of culled sows producing only one litter during their lifetime. U.S sow mortality increased from 8% in 2012 to 20.5% in 2023. These high culling and mortality rates reduce lifetime productivity, increase replacement costs, and erode profitability. This study identified factors associated with gilt retention at parities 1 and 3 in breeding herds under a single production system in the Midwestern United States. The dataset comprised 111,918 gilts (PIC 1050 genetics) bred in 2022. Thirteen variables were evaluated, including breeding sites, age and weight at first service, structural information, PRRSV, *Mycoplasma hyopneumoniae*, and Senecavirus A. Mixed-effects logistic regression models were fitted with sow farm as a random effect. Model building involved manual stepwise forward selection; interactions and confounders were tested based on biological plausibility. Pairwise comparisons used t-tests with Tukey-Kramer adjustment (*P* < 0.05). Across 55 farms, 69.2% of gilts were bred in gilt development units (GDU) and 30.8% in sow farms. Retention declined from 95% at parity 1 to 60% by parity 3. Main removal reasons were unspecified (27.6%), sudden death (15.5%), and lameness (12.1%), with sudden death most common at parity 1 (23.5%) and 2 (13.7%). Retention at parity 1 and 3 was associated with age at first service, PRRSV lifetime status, breeding site, and filtration type. Gilts bred under 28 to 33 weeks had 3% higher retention at parity 1 and 9% higher at parity 3 than gilts bred over 34 weeks (*P* < 0.001). Compared with gilts first bred at 28 to 33 weeks, parity 3 retention decreased by 6%, 11%, and 19% for gilts first bred at 34 to 36, 37 to 39, and >39 weeks, respectively. PRRSV-stable lifetime status herds had 12% higher retention at parity 3 (*P* < 0.001) than those that experienced an outbreak. Breeding gilts at GDU was associated with 27% higher retention rate at parity 3 compared to breeding in the sow farm (*P* < 0.001). Filtered farms had 2% higher retention rate at parity1 and 3 (*P* = 0.02). These results highlight the cumulative impact of reproductive management and herd environment on sow longevity. Optimizing gilt entry conditions, particularly PRRSV stability, age at first service, and breeding site, is a practical strategy for enhancing productivity and sustainability of breeding herds.

## Teaser

Age at first service, PRRS herd stability, and use of gilt development units were key drivers of sow retention through parity 3. Optimizing these gilt entry conditions offers a practical path to improving sow longevity, welfare, and breeding herd profitability.

## Introduction

Breeding herd profitability and sustainability hinge on optimizing individual sow performance and retention rates. Historically, many U.S. swine operations report annual replacement rates exceeding 50% and average parity at removal of only 3.5 to 3.8, with fewer than 40 pigs weaned per sow lifetime (Gruhot et al. 2017; Bergman et al. 2018; Koketsu et al. 2020). Sow retention represents a critical determinant of overall herd profitability and sow lifetime productivity (Patterson and Foxcroft 2019; Moeller and Stalder 2024). Many sows are prematurely replaced before their third parity, of which 15% to 20% of culled sows produce only one litter of pigs (Engblom et al. 2016, Stalder et al. 2004). Additionally, the United States has observed a rise in sow mortality, from 8% in 2012 to 20.5% in 2023 (Eckberg 2022; Paiva et al. 2023; MetaFarms 2024). Notably, this mortality is not evenly distributed across the breeding herd but is concentrated in younger females (12% in parity 0): the median parity at death has been reported as two, with most unplanned removals occurring in parity 0 to 1 sows (Kikuti et al. 2023). This concentration of mortality in early-parity females is particularly relevant, as these animals are removed before reaching parity 3, the parity at which a positive return on the replacement investment is typically achieved. This represents a significant opportunity to provide a conducive environment to reach their full production potential. Such high turnover levels inflate gilt development costs, reduce sows’ ability to produce multiple litters, and increase unit production costs. Sow removal patterns and reasons for removal have been linked to substantial economic loss and welfare concerns, underscoring the need to move from reactive culling toward proactive, data-informed management (Gruhot et al. 2017; Paiva et al. 2023; Ordaz et al. 2024). Benchmarking data indicate that the greatest attrition occurs between parity one and parity two, with reproduction failure accounting for approximately 39% of removals and lameness for 15.5% (Bergman et al. 2018; Patterson and Canavate 2023; Ordaz et al. 2024).

There are many time points in a gilt/sow production life cycle where these factors can positively or negatively impact retention (Patterson and Foxcroft 2019). Multiple studies have consistently identified actionable sow-level predictors of lifetime performance that can be monitored from gilt entry through later parities (Gruhot et al. 2017; Bergman et al. 2018; Koketsu et al. 2020; Ordaz et al. 2024). Gilt birth weight, age at first service, weight at breeding, and body condition changes are well-established predictors of sow lifetime productivity (Bortolozzo et al. 2009; Patterson and Foxcroft 2019; Iida et al. 2015; Faccin et al. 2022). Specifically, low-birth-weight gilts (<1.0 kg) exhibit impaired reproductive development and produce fewer piglets across parities compared to heavier contemporaries (Magnabosco et al. 2016; Patterson et al. 2018, 2020; Faccin et al. 2022). Age at first service is associated with improved subsequent fertility, longevity, and total lifetime production (Tani et al. 2018; Koketsu et al. 2020; Iida et al. 2015). Dedicated gilt development units (GDUs) have been recommended to standardize selection, nutrition, and breeding management practices, thereby narrowing the gap between genetic potential and realized lifetime productivity (Patterson and Foxcroft 2019).

Beyond reproductive metrics, health-related factors exert substantial influence on sow retention yet remain comparatively under-quantified in lifetime productivity models. Locomotor disorders, including lameness, are consistently among the top reasons for involuntary removal in U.S. and European systems, with reported within-herd lameness prevalence ranging from 9% to over 30% (Anil et al. 2009; Pluym et al. 2013; Nalon and Stevenson 2019; Paiva et al. 2023). PRRSV remains the most economically damaging pathogen affecting U.S. breeding herds (Osemeke et al. 2025), with unstable farms experiencing elevated abortion rates, increased preweaning mortality, and persistent reproductive disruption that extends culling pressure well beyond the acute outbreak phase (Holtkamp et al. 2013; Linhares et al. 2014; Sanhueza et al. 2019). *Mycoplasma hyopneumoniae*, although often framed as a grow-finish concern, has been associated with reduced lifetime sow performance in herds with active circulation, and its control status is increasingly used as a herd health benchmark in U.S. systems (Maes et al. 2008; Pieters and Maes 2019). More recently, Senecavirus A has emerged as a clinically relevant pathogen in breeding herds, producing vesicular lesions that complicate routine sow handling, breeding, and welfare assessment (Leme et al. 2015; Buckley 2021).

Retention is further shaped by herd-level characteristics that operate independently of, and interact with, individual sow attributes. Herd parity structure is both an outcome and a driver of retention: herds skewed toward low parities exhibit reduced piglets weaned per sow per year and greater susceptibility to disease incursion (Sasaki and Koketsu 2008; Iida et al. 2015). Breeding site, herd size, replacement gilt source, gilt acclimation protocols, and the disease status of the receiving herd have each been associated with measurable differences in removal risk and lifetime productivity (Koketsu 2007; Iida and Koketsu 2014; Patterson and Foxcroft 2019). Retention directly shapes herd parity distribution, contributing to a more balanced age structure that supports reproductive efficiency and productivity. It also contributes to herd health stability by minimizing turnover-related disease risks and enhances system sustainability through improved lifetime performance and more efficient resource utilization. However, substantial attrition persists, with only approximately 30% of gilts bred remaining in the herd through parity six (Patterson and Canavate 2023). Improving retention optimizes parity structure, increases pigs weaned per sow per year, and reduces replacement costs (Rodriguez-Zas et al. 2006; Gruhot et al. 2017). Reproductive and health predictors of sow longevity have been independently characterized in previous studies, yet their joint contribution to retention outcomes remains undefined. Therefore, the objective of this study was to identify factors associated with gilt retention at parity 1 and 3 in breeding herds under a single production system in the Midwestern United States.

## Materials and methods

### Overview

This retrospective observational study used data of gilts bred in 2022 from 55 farms in one commercial production system in the Midwestern United States. Integrated data from the gilt first service system, animal removal system, health data, and farm structural information were collated from the farm’s internal production system software (Iowa Select Farms TOOLS, Iowa Falls, IA). Figure 1 illustrates the data sources used to construct the master data file for the analysis. Table 1 shows a description of the variables used.

**Figure 1:**
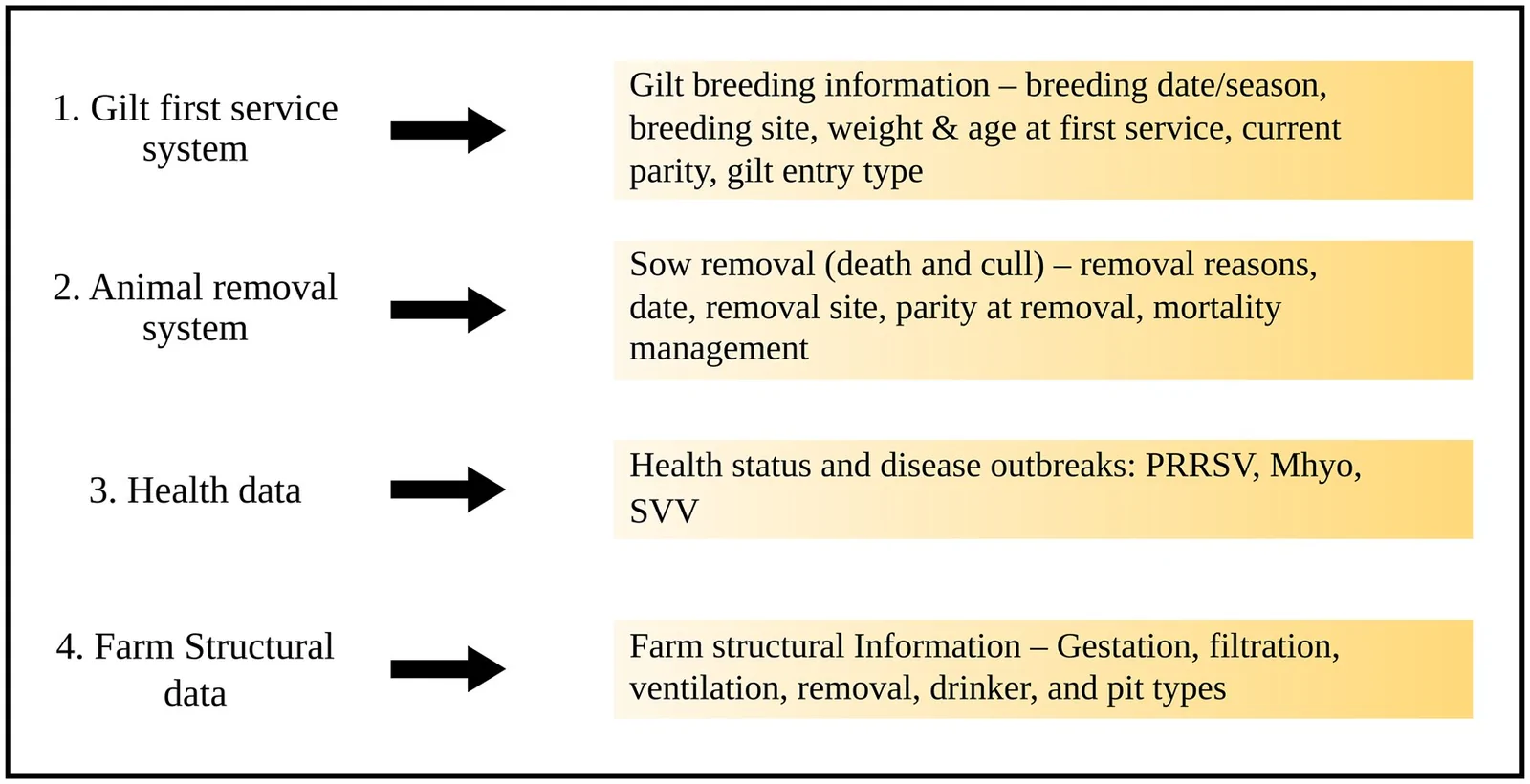
The different data sources used to build the final dataset. (1) Gilt first service system; (2) animal removal system; (3) health data; (4) farm structural data.

**Table 1:** Description of variables in the dataset.

Data source	Variables	Categories
**Breeding and management-related variables**	Breeding site	Sow farm, GDU
	Age at first service	Young (<28 weeks), Prime (28–33 weeks), Mature (34–36 weeks), Older (37–39 weeks), Late (>39 weeks)
	Weight at first service	<297 lbs (<135 kgs), Recommended 297–330 lbs (135–150 kgs), >330 lbs (>150 kgs)
	Removal type	Composting, Rendering
**Health variables**	PRRSV lifetime status	Outbreak, Consistently endemic, Stable
	Mycoplasma status	Positive, Negative
	SVA status	Positive, Negative
**Structural variables**	Gestation housing	Pens, Stalls
	Pit type	Deep, Shallow
	Farm air filtration type	Filtered, Non-filtered
	Drinker type	Nipple, Trough
	Ventilation	Negative, Positive pressure

### Data sources

#### Gilt first service system

Gilt breeding data were obtained from routine production records and included key variables relevant to reproductive performance and longevity. Specifically, information captured at first service included the breeding site, defined as a gilt development unit or a sow farm, flank-to-flank tape-measured body weight at service (Iwasawa et al. 2004), and age at first service grouped into: Young (<28 weeks), Prime (28 to 33 weeks), Mature (34 to 36 weeks), Older (37 to 39 weeks), and Late (>39 weeks). In addition, longitudinal records provided the current parity of each female, allowing for assessment of retention across successive parities.

#### Animal removal system

Animal removal data were extracted from the herd management recording system and included detailed information on sow removals throughout the production cycle. For each removal event, the dataset captured the primary reason for removal, the removal date, parity at removal, and the site where the removal occurred.

#### Health status classification

The health data consisted of assigning weekly disease herd status for PRRSV, *Mycoplasma hyopneumoniae* (Mhyo), Senecavirus A (SVA), and porcine epidemic diarrhea virus (PEDV) based on clinical signs, changes in performance, and routine laboratory testing performed in the herds. PRRSV and Mhyo weekly disease status was adapted by the production system from classifications currently adopted in the U.S. (Clavijo et al. 2021; Holtkamp et al. 2021). PRRSV lifetime status was categorized as outbreak, consistently endemic, or PRRSV stable (Paiva et al. 2023). PRRSV lifetime status reflected the PRRSV classification from the gilt’s entry into the breeding herd through her first farrowing (parity 1) and third farrowing (parity 3). The outbreak status was defined as the occurrence of wild-type PRRSV infection at any point during a gilt’s lifetime and was confirmed by diagnostic testing. In contrast, consistently endemic status referred to periods of low PRRSV prevalence with continued weekly surveillance using reverse transcription real-time polymerase chain reaction (RT-rtPCR) testing of processing fluid samples, while PRRSV-stable herds were classified as those in which gilts were in vaccinated herds, with consecutive weeks of negative RT-rtPCR results in the absence of clinical signs.

*Mycoplasma hyponeumoniae* and SVA status were defined according to Paiva et al. (2023). Herds were classified as Mhyo-negative when no compatible clinical signs were observed and routine diagnostic testing yielded negative results; otherwise, they were considered positive. Weekly SVA status was determined using analogous criteria: each week was classified as positive or negative based on the presence of clinical signs consistent with infection and laboratory confirmation of pathogen detection by RT-qPCR.

#### Structural information

Farm-level structural data included gestation housing systems (open-pen gestation or stall), air filtration status (filtered or unfiltered), pit type (deep or shallow), drinker types (nipple or trough) and mortality management (compost or rendering). These attributes were used to characterize the production environment and management practices at each site and were matched to either breeding or the current site.

### Study population and selection criteria

The unit of analysis for this study was the individual gilt. The study population comprised gilts bred in 2022 from a single genetic line (PIC 1050; PIC, Hendersonville, TN) with complete records for age and estimated body weight at first service. The final aggregated dataset comprised 111,918 gilts. Gilts with an age at first service of less than 25 weeks or greater than 47 weeks, as well as those with a body weight at first service below 265 lbs (120 kgs) or above 480 lbs (218 kgs), were excluded from the analysis. Additionally, gilts bred off-site were excluded from the analysis. Off-site breeding was defined as any gilts bred at sites other than the sow farm and the gilt development unit (GDU). Replacement gilts originated from a single multiplication source and entered commercial sow farms through one of two pathways. In the first pathway, gilts were transferred from the multiplier to a dedicated gilt development unit, where acclimation, boar exposure and breeding occurred and subsequently moved to the sow farm. In the second pathway, gilts were transferred directly to the sow farm, where breeding was conducted on site. Retention at parity 1 and 3 was defined as gilts that successfully farrowed at parity 1 and 3, respectively.

### Statistical analysis

#### Regression models and outcomes

Mixed logistic regression models were fitted to assess factors associated with retention at parity 1 and 3. A total of 13 variables were evaluated in each model, and the current site was included as a random effect to account for variations across different sow farms. Current site was defined as the site where the female was retained or removed post-first breeding in subsequent parities. All regression models were built in R (R Core Team, 2023, Vienna, Austria). The model-building process across the two outcomes used a manual, stepwise forward selection approach, in which interactions and confounders were tested based on biological relevance. Pairwise comparisons were tested using *t*-tests with the Tukey-Kramer adjustment; *P*-values at α 0.05 were considered statistically significant in the multivariable analysis.

#### Univariate analysis

Univariate analysis was performed on all explanatory variables to determine their association with the two outcomes independently. Variables that yielded a *P*-value of ≤ 0.20 were considered for inclusion in the subsequent multivariable modeling (Dohoo et al. 2009). Prior to multivariable analysis, the explanatory variables were assessed for pairwise correlations using the Variance Inflation Factor (VIF) to detect potential multicollinearity. Variables were considered collinear if the VIF exceeded 5.

#### Multivariable analysis

The multivariable model-building process involved a manual stepwise forward selection approach with biological plausibility (Dohoo et al. 2009). Breeding season was included in the model to adjust for season-to-season variations. Variables that showed potential associations in the univariate analysis (*P* ≤ 0.20) were sequentially added to the model based on their statistical significance and biological plausibility. At each step, the contribution of the variable to the model was assessed using likelihood ratio tests, and only those variables that significantly improved model fit (*P* < 0.05) was retained. Potential confounding variables were evaluated by examining changes in the estimated coefficients of primary explanatory variables upon their inclusion. A variable was considered a confounder if its inclusion altered the coefficient of another variable by more than 20%. Biologically plausible interaction terms and pairwise comparisons were tested using *t*-tests with the Tukey-Kramer adjustment; *P*-values <0.05 were considered statistically significant. Estimated marginal means were computed on the logit scale and back-transformed to the probability scale using the inverse logit function. Retention probabilities and their 95% confidence intervals (CI) were reported as percentages.

## Results

### Overview

Table 1 presents the variables used in univariate and multivariable analyses. Across the 55 farms included in the study, a total of 111,918 animals were represented. GDU sites comprised 77,484 animals (69.2%) from 19 farms, whereas sow farms comprised 34,434 animals (30.8%) from 36 farms. Overall retention declined from 95% at parity 1 to 60% by parity 3. Among sows that were removed, the leading reasons were unspecified (27.6%), sudden death (15.5%), lameness (12.1%), poor body condition (9.6%), prolapse (8.8%), reproductive reasons (8.1%), farrowing issues (6.9%), anestrus (4.7%), downer (3.9%), and depopulation (2.8%). Sudden death was the most common removal reason in Parity 1 (23.5%) and Parity 2 (13.7%). Removal reasons by age and estimated weight at first service are presented in Tables 4 and 5 respectively. Lameness accounted for a similar proportion of removals in gilts bred above 330 lbs (10.1%) and in those bred at 297 to 330 lbs (11.8%) in this dataset. Gilts bred below 297 lbs accounted for only five removals (0.6%), three of which were attributed to lameness. These percentages represent the distribution of causes among removed animals and are therefore calculated relative to the removed population. A summary of factors associated with gilt retention, presented in univariate and multivariable analyses, is shown in Tables 2 and 3.

**Table 2:** Factors associated with retention at parity 1 and 3 from univariate analysis.

Outcomes	Factors	Categories (levels)	LS Means Retention (%)^*^	*P*-value
**Retention at parity 1**	Breed site	GDU	96.8^a^	0.001
		Sow farm	90.1^b^	
	Age at first service	Young (<28 weeks)	96.4^a^	<0.001
		Prime (28–33weeks)	95.9^a^	
		Mature (34–36 weeks)	93.5^b^	
		Older (37–39 weeks)	92.0^bc^	
		Late (>39 weeks)	90.0^c^	
	Weight at first service (lbs)	< 297 lbs (<135kgs)	91.9^b^	0.010
		Recommended 297–330 lbs (135–150 kgs)	95.6^a^	
		> 330 lbs (>150 kgs)	94.7^a^	
	Filtration type	Non-Filtered	95.4^b^	<0.001
		Filtered	96.6^a^	
	PRRSV lifetime status	Outbreak	93.8^b^	
		Consistently endemic	94.9^a^	<0.001
		No outbreak (stable)	96.3^a^	
	Gestation type	Pens	95.1^a^	<0.001
		Stalls	91.3^b^	
	Removal type	Composting	96.1^a^	0.022
		Rendering	94.2^b^	
	Ventilation	Negative Pressure	92.2^a^	<0.001
		Positive Pressure	95.5^b^	
	Drinker type	Nipple	93.7^a^	<0.001
		Trough	87.8^b^	
		Water-tree	93.6^a^	
		Wet-dry	95.4^c^	
	Pit type	Deep	95.8^a^	<0.001
		Shallow	92.9^b^	
	Breeding Season	Fall	94.7^ab^	0.053
		Spring	94.9^a^	
		Summer	94.5^ab^	
		Winter	94.0^b^	
	SVA status	Positive	94.1^b^	<0.001
		Negative	93.0^a^	
**Retention at parity 3**	Breed site	GDU	50.5^a^	0.001
		Sow farm	46.8^b^	
	Age at first service	Young (<28 weeks)	63.0^a^	<0.001
		Prime (28–33 weeks)	58.8^a^	
		Mature (34–36 weeks)	51.2^b^	
		Older (37–39 weeks)	46.5^c^	
		Late (>39 weeks)	36.6^d^	
	Weight at first service (lbs)	< 297 lbs (<135 kgs)	47.3^ab^	0.02
		Recommended 297–330 lbs (135–150 kgs)	49.9^b^	
		> 330 lbs (>150 kgs)	48.5^a^	
	Filtration type	Non-Filtered	40.0^b^	0.03
		Filtered	42.0^a^	
	PRRSV lifetime status	Outbreak	37.9^b^	
		Consistently endemic	47.2^c^	<0.001
		No outbreak(stable)	60.7^a^	
	Gestation type	Pens	62.2^a^	<0.001
		Stalls	39.7^b^	
	Removal type	Composting	70.8^a^	<0.001
		Rendering	60.1^b^	
	Ventilation	Negative Pressure	50.2^a^	<0.001
		Positive Pressure	62.9^b^	
	Drinker type	Nipple	67.9^a^	<0.001
		Trough	48.4^b^	
		Water-tree	55.0^c^	
		Wet-dry	58.8^d^	
	Pit type	Deep	62.8^a^	<0.001
		Shallow	56.3^b^	
	Breeding Season	Fall	55.2^a^	<0.001
		Spring	63.4^b^	
		Summer	59.1^c^	
		Winter	61.9^d^	

* Superscript letters indicate statistical significance at *P* < 0.050 between levels within factors.

**Table 3:** Factors associated with retention at parity 1 and 3 from multivariate analysis.

Factors	Categories (levels)	LS Means Retention (%)	95% CI*	*P*-value
**Retention at parity 1**				
**Breed site**	GDU	95.0^a^	94.8–95.2	0.05
	Sow farm	92.1^b^	90.9–93.1	
**Age at first service**	Young (<28 weeks)	96.0^a^	94.6–97.0	<0.001
	Prime (28–33 weeks)	94.9^a^	94.8–95.2	
	Mature (34–36 weeks)	93.0^b^	92.7–93.3	
	Older (37–39 weeks)	92.1^bc^	91.5–92.5	
	Late (>39 weeks)	91.0^c^	90.1–91.8	
**Filtration type**	Non-Filtered	93.0^b^	92.6–93.8	<0.001
	Filtered	95.2^a^	94.8–95.4	
**PRRSV lifetime status**	Outbreak	93.6^a^	93.3–93.9	0.04
	Consistently endemic	94.0^a^	93.6–94.3	
	No outbreak	95.0^b^	94.8–95.3	
**Retention at parity 3**				
**Breed site**	GDU	55.6^a^	43.7–60.6	<0.001
	Sow farm	27.8^b^	26.7–38.7	
**Age at first service**	Young (<28 weeks)	53.8^a^	50.3–57.6	<0.001
	Prime (28–33 weeks)	50.8^a^	48.9–51.6	
	Mature (34–36 weeks)	44.8^b^	44.2–45.8	
	Older (37–39 weeks)	39.6^c^	38.8–41.2	
	Late (>39 weeks)	31.9^d^	30.6–34.0	
**Weight at first service**	< 297 lbs (<135kgs)	39.3^a^	37.0–41.6	0.01
	Recommended 297–330 lbs	42.5^b^	41.8–43.2	
	> 330 lbs (>150kgs)	41.2^a^	40.6–41.8	
**Filtration type**	Non-Filtered	40.0^b^	39.1–41.6	0.03
	Filtered	42.0^a^	40.5–42.8	
**PRRSV lifetime status**	Outbreak	35.5^a^	34.9–36.1	<0.001
	Consistently endemic	39.8^b^	38.9–40.7	
	No outbreak	47.9^c^	46.8–48.6	

95% CI*, 95% Confidence Interval.

* Superscript letters indicate statistical significance at *P* < 0.050 between levels within factors.

**Table 4:** Removal reasons of sows by age at first service.

Removal reason	Age at first service (weeks)					Total
	<28	28–33	34–36	37–39	>39	
**Unspecified**	0 (0.0)	157 (37.7)	129 (44.2)	69 (45.1)	7 (46.7)	362 (41.3)
**Sudden death**	0 (0.0)	73 (17.5)	39 (13.4)	28 (18.3)	3 (20.0)	143 (16.3)
**Lameness**	0 (0.0)	46 (11.1)	37 (12.7)	12 (7.8)	2 (13.3)	97 (11.1)
**Prolapse**	0 (0.0)	35 (8.4)	16 (5.5)	8 (5.2)	0 (0.0)	59 (6.7)
**Downer**	0 (0.0)	23 (5.5)	17 (5.8)	9 (5.9)	1 (6.7)	50 (5.7)
**Poor body condition**	1 (100.0)	24 (5.8)	14 (4.8)	10 (6.5)	0 (0.0)	49 (5.6)
**Farrowing issues**	0 (0.0)	16 (3.8)	14 (4.8)	8 (5.2)	1 (6.7)	39 (4.4)
**Respiratory**	0 (0.0)	11 (2.6)	8 (2.7)	6 (3.9)	0 (0.0)	25 (2.9)
**Breeding failure**	0 (0.0)	14 (3.4)	9 (3.1)	1 (0.7)	0 (0.0)	24 (2.7)
**Abortion**	0 (0.0)	6 (1.4)	5 (1.7)	1 (0.7)	0 (0.0)	12 (1.4)
**Anestrus**	0 (0.0)	6 (1.4)	3 (1.0)	0 (0.0)	1 (6.7)	10 (1.1)
**Gastric ulcers**	0 (0.0)	3 (0.7)	0 (0.0)	1 (0.7)	0 (0.0)	4 (0.5)
**Depopulation**	0 (0.0)	2 (0.5)	1 (0.3)	0 (0.0)	0 (0.0)	3 (0.3)
**Total**	**1**	**416**	**292**	**153**	**15**	**877**

Values are n (column %). Removal reasons are ranked by total number of removals.

**Table 5:** Removal reasons of sows by weight at first service.

Removal reason	Estimated weight at first service			Total
	<297 lbs	297–330 lbs	>330 lbs	
**Unspecified**	1 (20.0)	113 (31.8)	248 (48.0)	362 (41.3)
**Sudden death**	0 (0.0)	65 (18.3)	78 (15.1)	143 (16.3)
**Lameness**	3 (60.0)	42 (11.8)	52 (10.1)	97 (11.1)
**Prolapse**	0 (0.0)	27 (7.6)	32 (6.2)	59 (6.7)
**Downer**	0 (0.0)	19 (5.4)	31 (6.0)	50 (5.7)
**Poor body condition**	1 (20.0)	28 (7.9)	20 (3.9)	49 (5.6)
**Farrowing issues**	0 (0.0)	17 (4.8)	22 (4.3)	39 (4.4)
**Respiratory**	0 (0.0)	9 (2.5)	16 (3.1)	25 (2.9)
**Breeding failure**	0 (0.0)	17 (4.8)	7 (1.4)	24 (2.7)
**Abortion**	0 (0.0)	8 (2.3)	4 (0.8)	12 (1.4)
**Anestrus**	0 (0.0)	6 (1.7)	4 (0.8)	10 (1.1)
**Gastric ulcers**	0 (0.0)	2 (0.6)	2 (0.4)	4 (0.5)
**Depopulation**	0 (0.0)	2 (0.6)	1 (0.2)	3 (0.3)
**Total**	**5**	**355**	**517**	**877**

Values are *n* (column %). Removal reasons are ranked by total number of removals.

### Univariate analysis

In the univariate analyses, several management, disease-related, and farm structural variables were associated with gilt retention at parity 1 and parity 3 (Table 2). Among management factors, age at first service, body weight at first service, and breeding site were significantly associated with retention at both parity 1 and parity 3 (*P* < 0.05). Breeding season was marginally associated with retention at parity 1 (*P* = 0.05) but showed a significant association with retention at parity 3 (*P* < 0.001). PRRSV lifetime status, SVA status, gestation type, and filtration type were significantly associated with retention at parity 1 and parity 3 (*P* < 0.05).

### Multivariable analysis

The multivariable models identified four variables as statistically significant predictors of retention at parity 1 and five variables as statistically significant predictors of retention at parity 3, as shown in Table 3. The mixed-effects logistic regression showed substantial clustering by site, with an adjusted intraclass correlation coefficient (ICC) of 0.38, indicating that 38% of the variance in the model was attributable to differences between sow farms.

### Breeding site

At the first parity, gilts bred at GDU had 3% higher retention than those from a sow farm (*P* = 0.05) (Fig. 2). Breeding gilts at GDUs rather than sow farms was associated with a 27% increase in retention at parity 3 (*P* < 0.001).

**Figure 2:**
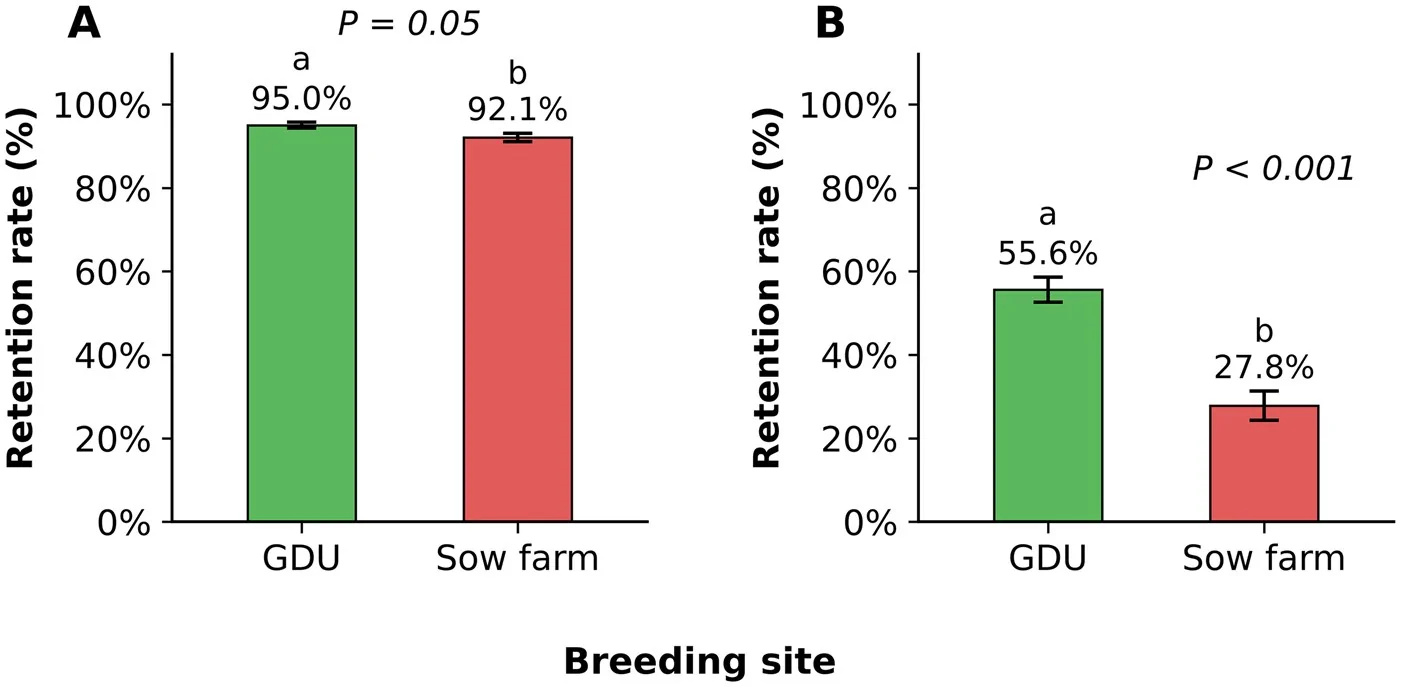
Factors associated with gilt retention at parity 1(A) and parity 3 (B). The plots show the impact of breeding sites on retention rates at parities 1 and 3. The bars represent the least-squares (LS) means of retention rate (%) for each breeding site category, with error bars indicating the 95% upper confidence intervals. The letters above the bars indicate significant differences among groups (*P* < 0.05).

### Age at first service

Gilt age at first service was negatively associated with retention at both parity 1 and parity 3, with the decline more pronounced at later ages (Fig. 3). Gilts bred below 28 to 33 weeks of age had 3% higher retention at parity 1 (*P* < 0.001) and 9% higher retention at parity 3 compared to gilts bred after 34 weeks (*P* < 0.001). Retention decreased further by 12.9% for gilts bred after 39 weeks compared to those bred between 28 and 33 weeks at parity 3. At parity 1, gilts bred at <28 weeks retained 5% more than those bred at >39 weeks (*P* = 0.01), indicating statistically significant but relatively modest divergence at this early stage. Retention rate at parity 3 was 19% higher in gilts bred at 33 weeks compared to those bred at >39 weeks (*P* < 0.001). Relative to gilts bred at 28 to 33 weeks, retention at parity 3 was reduced by 6%, 11%, and 19% for gilts bred at 34 to 36, 37 to 39, and >39 weeks, respectively, demonstrating a cumulative negative effect of delayed breeding. Notably, retention for gilts bred after 36 weeks decreased to 13% by parity 3, highlighting the substantial impact of late breeding on sow longevity. The interaction of age and weight at first service was not statistically significant at parity 1 (*P* = 0.25) and parity 3 (*P* = 0.42).

**Figure 3:**
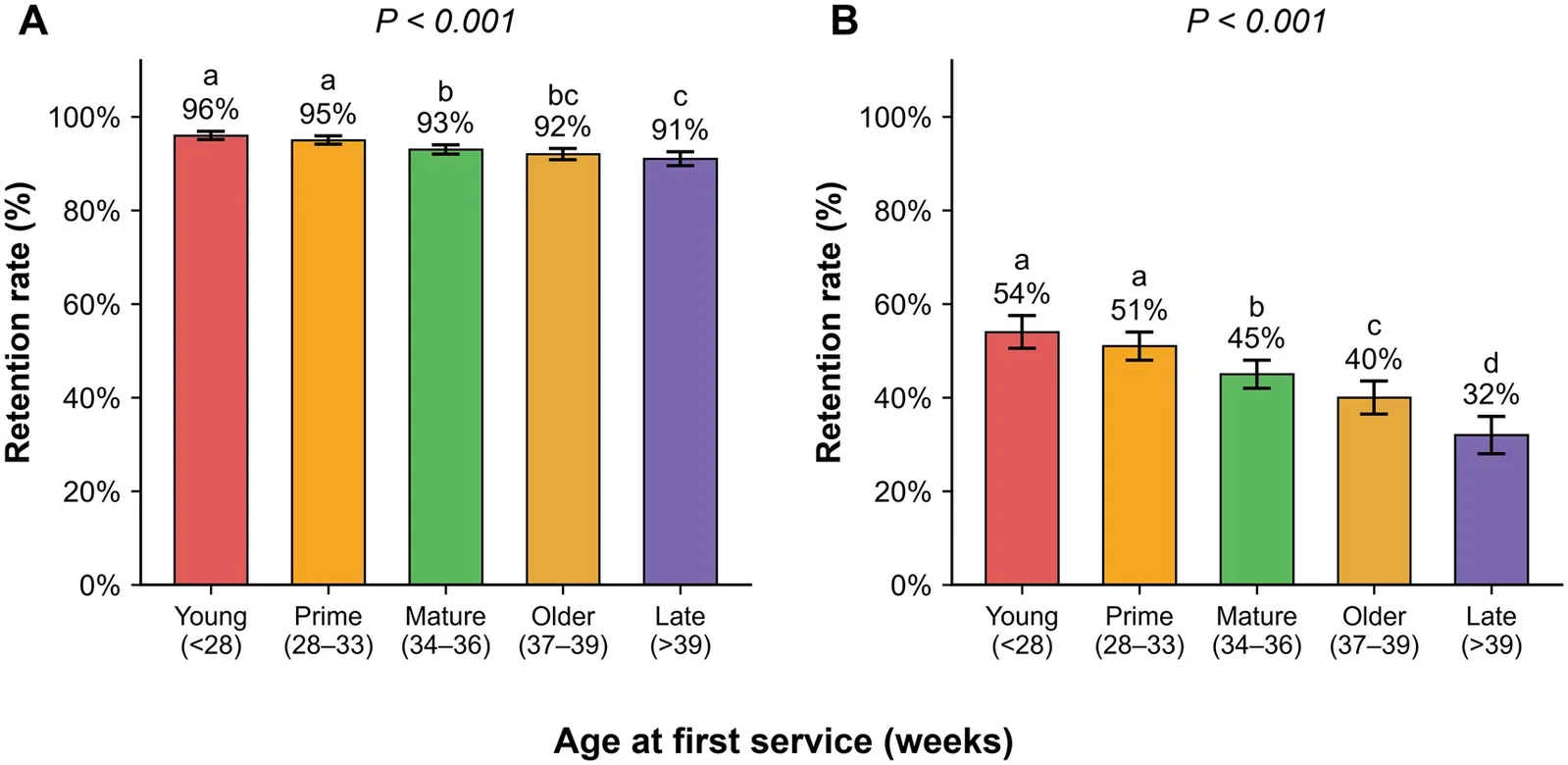
Factors associated with gilt retention at parity 1(A) and parity 3 (B). The plot shows the impacts of age at first service on retention rates at parities 1 and 3. The bars represent the least-squares (LS) means of retention rate (%) for each age-at-first-service category, with error bars indicating the 95% upper confidence intervals. The letters above the bars indicate significant differences among groups (*P* < 0.05).

### PRRSV lifetime herd status

Gilts in PRRSV-stable herds had greater retention than gilts in outbreak and consistently endemic herds (Fig. 4). At parity 1, retention differed by PRRSV lifetime status (*P* = 0.01). Gilts in stable herds had higher retention (95.0%) than gilts that experienced an outbreak (93.6%) and gilts in consistently endemic herds (94.0%), differences of 1.4% and 1.0%, respectively. Retention at parity 1 did not differ between outbreak and consistently endemic herds. At parity 3, the differences in retention rate were more pronounced (*P* < 0.001). Gilts in stable herds had 47.9% retention, which was 12.4% higher than gilts that experienced an outbreak between first breeding and parity 3 (35.5%) and 8.1% higher than gilts in consistently endemic herds (39.8%). Gilts consistently endemic herds had 4.3% higher retention than gilts that experienced an outbreak.

**Figure 4:**
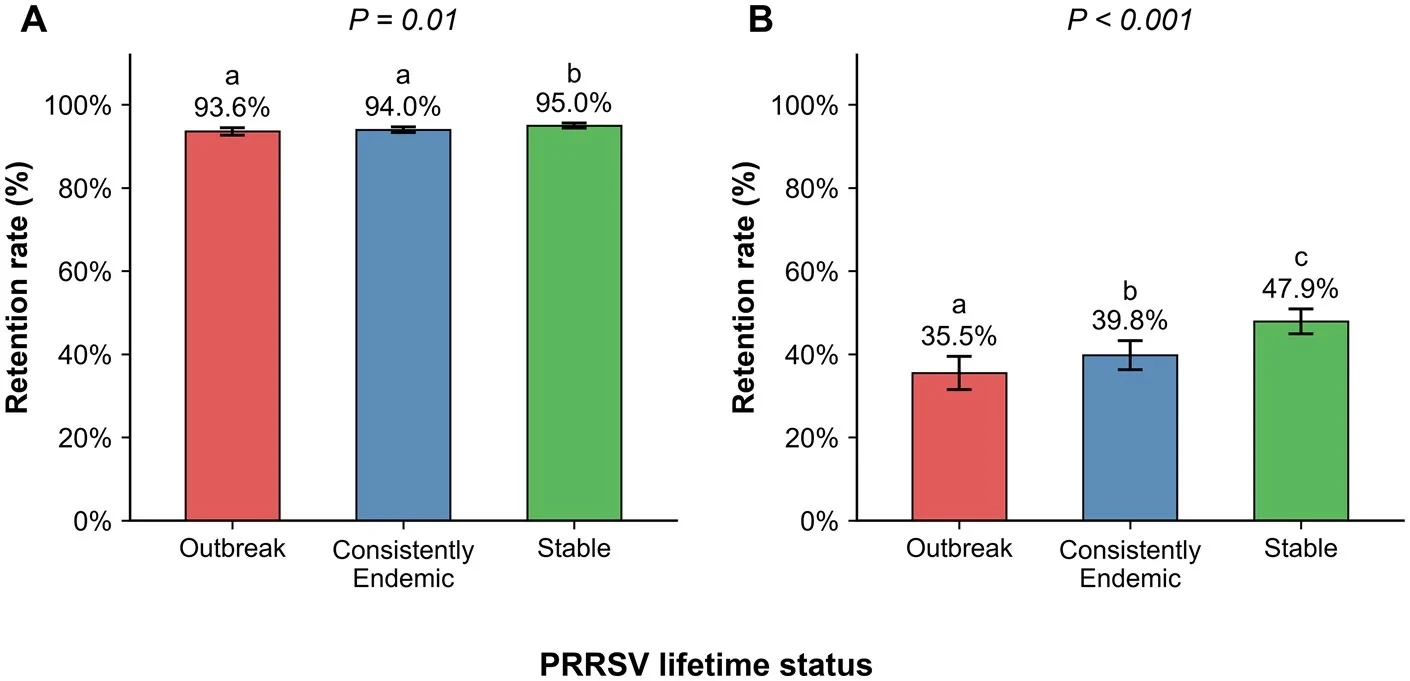
Factors associated with gilt retention at parity 1(A) and parity 3 (B). The plots show the impact of PRRSV lifetime status on retention rates at parities 1 and 3. The bars represent the least-squares (LS) means of retention rate (%) for each PRRSV lifetime status category, with error bars indicating the 95% upper confidence intervals. The letters above the bars indicate significant differences among groups (*P* < 0.05).

### Estimated weight at first service

Estimated weight at first service was a statistically significant variable in parity 3 (*P* = 0.01) but was not statistically significant at parity 1. The results are summarized in Fig. 5.

**Figure 5:**
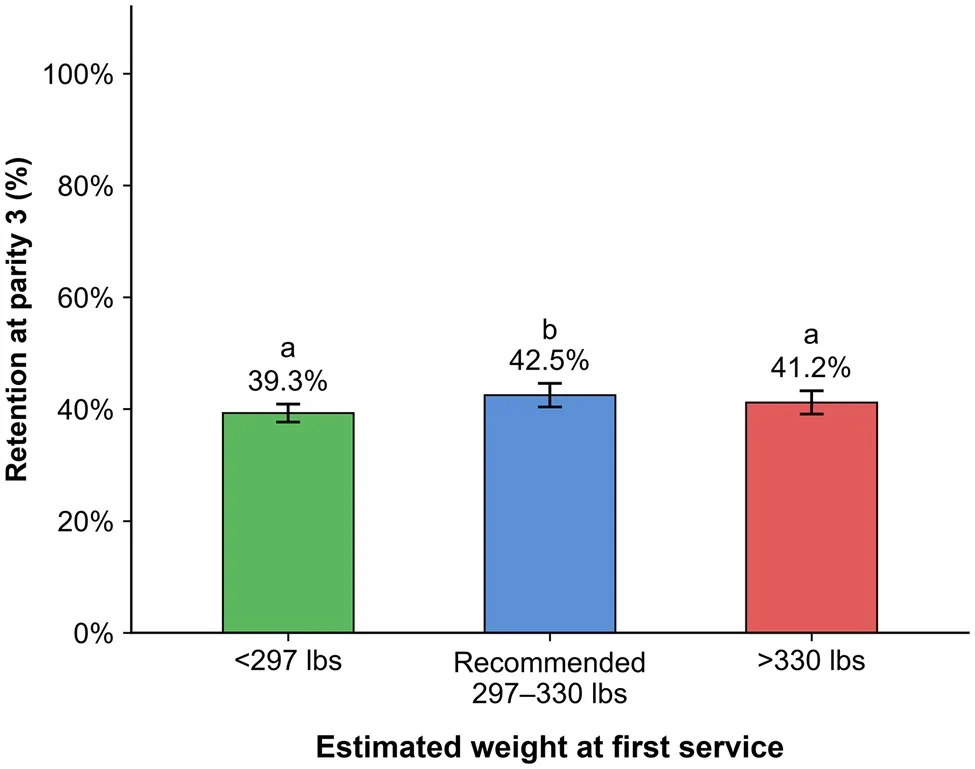
The plot shows the impact of estimated weight at first service on retention rates at 3. The bars represent the least-squares (LS) means of retention rate (%) for each estimated weight category, with error bars indicating the 95% upper confidence intervals. The letters above the bars indicate significant differences among groups (*P* < 0.05).

### Filtration status

Filtered farms were associated with 2.2% higher retention rate at parity 1 (*P* < 0.001) and 2% higher retention rate at parity 3 (*P* = 0.03) than non-filtered farms, after adjustment for PRRSV lifetime status (Table 3).

## Discussion

The results of this analysis indicate that gilt retention is influenced by multiple factors, including age and weight at first service, lifetime PRRSV status, farm filtration status, and breeding site. All variables independently demonstrated statistically significant associations with retention at univariate analysis, with substantially amplified effects at parity 3 compared to parity 1. This parity-dependent effect indicates that management decisions implemented during the critical gilt development phase have compounding consequences that gradually manifest over successive reproductive cycles. In the multivariable models, breed, site, age at first service, filtration type, and PRRSV lifetime status were associated with retention to parity 1, whereas these variables and weight at first service were associated with retention to parity 3. Although weight at first service was associated with retention in the univariable analyses at both parities, the association remained significant only for retention to parity 3 in the multivariable model. This parity-specific association may suggest that the relationship between body weight at breeding and subsequent sow retention becomes more apparent beyond the first parity. The adjusted ICC (0.38) indicates considerable between-farm variation in retention to parity 1 and parity 3. Therefore, factors operating at the farm level may contribute to variation in gilt retention beyond the individual-level factors evaluated. Premature removal of gilts before reaching economically productive parities represents a substantial opportunity loss, as the cumulative genetic and physiological investments made during gilt development are unrealized (Joab 2019; Patterson and Foxcroft 2019; Lucia 2024).

Breeding gilts at GDUs rather than sow farms was associated with a 27% increase in retention at parity 3. The pronounced difference may be attributed to management and structural benefits of dedicated GDUs, controlled acclimation, and standardized management practices, which have cumulative positive effects on gilt health and reproductive performance, resulting in higher long-term retention than gilts bred directly on sow farms. This substantial effect persists after adjustment for other management and health variables. Patterson and Foxcroft (2019) reported that the routine implementation of efficient gilt development units (GDUs) delivers high-quality, breeding-eligible gilts to sow farms. Additionally, optimal gilt management begins at birth, as litter of origin, lactation management, and early gilt selection strategies serve as early indicators of future performance and efficiency (Koketsu et al. 2020; Carrión-López et al. 2022; Faccin et al. 2022). The higher retention in gilts bred in the GDUs may reflect multiple complementary factors in this production system. First, dedicated gilt-specific management protocols that avoid competitive disadvantages with mature sows for housing space, feeding access, and social resources. Secondly, standardized nutrition and growth management targeted at achieving optimal body composition for reproduction, including controlled pathogen acclimation under defined biosecurity conditions. Additionally, this may reflect improved gilt selection, enhanced boar exposure, and enhanced estrus synchronization procedures. Faccin et al. (2022) demonstrated that managing replacement gilts to achieve optimal body weight and growth rates during the boar stimulation phase and pre-breeding period represents a critical component of sow reproductive longevity and producer profitability.

Our findings indicate that delayed age at first service was associated with reduced retention through parity 3. Gilts bred beyond 39 weeks of age had a 19% lower parity 3 retention rate than gilts first bred at 28 to 33 weeks of age. This reduction was incremental, with retention decreasing by 6% and 11% among gilts first bred at 34 to 36 and 37 to 39 weeks, respectively. The progressive inverse relationship between age at first service and sow retention is consistent with prior literature linking delayed breeding to compromised gilt reproductive longevity (Stalder et al. 2004; Hoge and Bates 2011; Patterson and Foxcroft 2019; Koketsu et al. 2020; Aguirre et al. 2025). The mechanisms underlying this relationship are multifaceted. Saito et al. (2011) reported that as age at first mating increased from 209–229 days to 272–365 days, annualized lifetime pigs born alive decreased from 18.2 to 15.3, and the number of parities at removal declined significantly. This longevity penalty likely reflects accumulated physiological delays and metabolic imbalances in older gilts. Lucia (2024) reported that gilts that exhibit first estrus at early ages accumulate fewer non-productive days prior to the first service and are more likely to have greater lifetime productivity than those reaching the first estrus at older ages. Delayed puberty in older gilts may reflect underlying metabolic dysfunction, impaired reproductive tract maturation, or diminished oocyte quality, thereby contributing to reduced fertility and an increased risk of culling. Older gilts (>250 days) at breeding are at substantially increased risk for premature culling, primarily due to reproductive failure and locomotor complications, with elevated mortality rates (Joab 2019; Belkova and Rozkot 2022; Faccin et al. 2022; Aguirre et al. 2025). The interplay between excessive age and concurrent overweight status exacerbates these risks synergistically. These findings provide empirical support for concentrated gilt development protocols targeting earlier breeding ages and reinforce industry benchmarks.

Previous literature indicates that delayed age at first mating is associated with reduced longevity and increased risk of reproductive failure, while excessive body weight at breeding has been associated with reduced sow lifetime productivity and increased early culling risk (Bortolozzo et al. 2009). These findings provide biological context for the associations observed in the present study, but the specific pathways leading to removal cannot be determined from the present data. In the study, gilts bred at 297 to 330 lbs (135 to 150 kg) had a higher retention rate at parity 3 than both lighter (<297 lbs) and heavier (>330 lbs) gilts. These findings are consistent with extensive literature on the critical importance of gilt weight at breeding (Bortolozzo et al. 2009; Carrión-López et al. 2022; Tan et al. 2023; Aguirre et al. 2025). Bortolozzo et al. (2009) reported that overweight gilts (>150 kg) face a higher risk of reduced longevity because sows with excess body weight are more likely to develop locomotor disorders. Carrión-López et al. (2022) reported that sows in the small body weight group (<135 kg) at first service numerically had one less parity across their lifetime compared to large sows (>150 kg) and tended to have a higher culling rate over three parities (Carrión-López et al. 2022).

However, the mechanism proposed by Bortolozzo et al. (2009) was not clearly reflected in the removal reasons recorded in the present study. Lameness accounted for a similar proportion of removals in gilts bred above 330 lbs (>150 kg) and in those bred at 297 to 330 lbs (135 to 150 kg). This suggests that the lower retention of heavy gilts in our study was not driven primarily by lameness. Instead, a larger share of removals in heavy gilts was recorded without a specified reason, which limits our ability to identify the causes underlying their reduced retention. Lameness may also be under-represented if some lame animals were recorded under unspecified, downer or poor body condition categories. More consistent and specific recording of removal reasons on commercial farms would help clarify how weight at breeding influences sow longevity.

The association between PRRSV lifetime status and gilt retention underscores the well-documented impact of PRRSV on sow herd productivity. In this study, gilts in PRRSV-stable herds were associated with higher retention than those exposed to outbreaks. Specifically, retention at parity 1 was 2.6% higher in stable herds compared with herds that experienced an outbreak. This difference widened by parity 3, where retention was 12.4% higher among gilts in stable herds than among those experiencing an outbreak between first breeding and parity 3. The increasing disparity across parities suggests a cumulative immunological and reproductive burden associated with repeated viral exposure over successive breeding cycles. The 12.4% difference in parity 3 retention highlights the longer-term consequences of acute PRRSV exposure during the productive lifespan of gilts. Additionally, our study illustrates that the consistently endemic herds exhibited an intermediate but distinct retention rate at parity 3 (39.8%), demonstrating that even sub-clinical enzootic PRRSV exerts a measurable toll on gilt longevity.

Papatsiros (2012) demonstrated that PRRSV-infected farms experience increased culling rates, primarily due to reproductive problems and accelerated culling of young females, with significant economic importance. Osemeke et al. (2025) reported that PRRSV is responsible for annual economic losses of $1.2 billion due to productivity losses across U.S. breeding and growing-pig populations, with losses driven substantially by reproductive failure and elevated culling in breeding-age females. Lunney et al. (2016) observed that gilts are markedly more susceptible to PRRSV than older sows and experience greater phenotypic disease impact, reinforcing why endemic viral circulation may disproportionately affect younger breeding females. The economic implications are substantial. Our findings provide empirical evidence that PRRSV status is associated with sow retention and longevity, highlighting an additional production consequence of PRRSV beyond its effects on immediate litter performance.

Filtered farms were associated with 2% higher retention rate at parity 1 and parity 3 than non-filtered farms, after adjustment for PRRSV lifetime status. The primary mechanism may be attributed to a reduction in airborne pathogen burden, particularly respiratory viruses such as PRRSV and swine influenza virus, and other pathogens transmitted via aerosol routes. Many swine farms across North America have adopted air filtration technologies, which have been associated with a marked reduction in the risk of PRRSV introduction (Spronk et al. 2010; Dee et al. 2012). Alonso et al. (2013) quantified this protective effect in a prospective 7-year study in swine-dense regions, reporting that air filtration reduced the risk of novel PRRSV introduction by approximately 80% and prevented approximately four-fifths of PRRSV outbreaks in herds with good biosecurity practices. Dee et al. (2012) provided evidence that air filtration provides durable multi-year protection against new viral introductions in swine-dense production regions, suggesting a sustained cumulative benefit. Sites without air filtration demonstrated substantially increased odds (20-fold) of experiencing PRRSV outbreaks, further supporting the protective mechanism.

The interrelated nature of identified risk factors suggests that optimal retention requires simultaneous attention to multiple developmental parameters rather than sequential optimization of individual factors. Age and weight at first service are intrinsically linked because they depend on growth rate and maturation timing. PRRSV stability enables proper immune development in young gilts, while dedicated GDU facilities create the management infrastructure necessary to simultaneously optimize these multiple parameters under standardized conditions. Barn filtration provides the biosecurity foundation enabling maintenance of the health status necessary for optimal reproductive performance. This integrated perspective suggests that maximum benefit will accrue when all factors are simultaneously optimized.

However, some limitations must be highlighted. This analysis was conducted within a single commercial production system with uniform genetics (PIC 1050), standardized management practices, and specific biosecurity protocols, which may limit generalizability to herds with different management systems, genetic backgrounds, or disease challenges. Additionally, the dataset, while substantial in sample size, represents a single production system located in a region of high pig density and disease challenge in the Midwestern United States, potentially limiting its applicability to other geographic regions with different disease prevalence and housing conditions. Future research involving diverse commercial production systems and prospective study designs would strengthen and extend these findings across the broader swine production industry.

## Conclusion

This multivariable analysis demonstrates that gilt retention through parity 3 is influenced by a combination of biological, management, and environmental factors, including age and weight at first service, lifetime PRRSV herd status, breeding site origin, and barn filtration infrastructure. Notably, the magnitude of these associations was greater at parity 3 than at parity 1, indicating that decisions made during the gilt development phase exert cumulative and progressively amplified effects on sow longevity across successive reproductive cycles. This pattern underscores the longitudinal nature of reproductive efficiency and the critical importance of early-life optimization. Collectively, these findings support the implementation of integrated, systems-based management strategies, applied concurrently rather than in isolation to enhance gilt retention, reduce involuntary culling, improve herd stability, and ultimately increase the long-term productivity, profitability, and sustainability of commercial breeding systems.

## List of abbreviations

GDU: Gilt Development Unit
PRRSV: Porcine Reproductive and Respiratory Syndrome virus
SVA: Senecavirus A
PIC: Pig Improvement Company
IA: Iowa
TN: Tennessee
WI: Wisconsin
α: probability of type I error
CI: confidence interval
